# Genetic Analysis of Superoxide Dismutase-1 (SOD1) +35A/C (exon 3/intron 3) and 50-bp Insertion/Deletion Polymorphisms in Synthetic Cannabinoid Use Disorder and Their Clinical Relevance

**DOI:** 10.1192/j.eurpsy.2026.10508

**Published:** 2026-07-31

**Authors:** H. M. Aytac, M. Pehlivan, Y. Oyaci, E. Aytac, S. Pehlivan

**Affiliations:** 1 Institute of Graduate Studies in Health Sciences, Istanbul University; 2 Department of Psychiatry, University of Health Sciences; 3 Department of Hematology, Basaksehir Cam and Sakura City Hospital; 4 Department of Medical Biology, Istanbul University, Istanbul; 5 Department of Psychiatry, Konya Beyhekim Training and Research Hospital, Konya, Türkiye

## Abstract

**Introduction:**

Superoxide dismutases (SODs) are key antioxidant enzymes that neutralize superoxide radicals. Mammals express three isoforms: SOD1, SOD2, and SOD3. SOD1, a cytosolic copper-zinc enzyme encoded on chromosome 21q22, detoxifies reactive oxygen species. The gene has five exons and four introns, with notable variants including a 50 bp promoter Ins/Del and the +35A/C (rs2234694) polymorphism, both linked to various clinical conditions. Their role in synthetic cannabinoid use disorder (SCUD), however, remains unexplored. We hypothesize that these *SOD1* variants may influence susceptibility to SCUD.

**Objectives:**

This study aims to investigate the relationship between clinical features of SCUD and two genetic variants of the *SOD1* gene: the +35A/C (rs2234694) and the 50 bp insertion/deletion (Ins/Del) polymorphisms. Specifically, we sought to compare the genotype distributions of these polymorphisms between patients with SCUD and healthy controls, and to explore their potential associations with clinical characteristics.

**Methods:**

This case-control study included 168 patients with SCUD and 150 age- and sex-matched healthy controls. The *SOD1* rs2234694 (+35A/C) and 50-bp insertion/deletion (Ins/Del) polymorphisms were analyzed using PCR-RFLP. For the +35A/C variant, DNA samples were amplified under standard PCR conditions, digested with HhaI, and visualized on agarose gel: AA (278 bp), AC (278, 207, 71 bp), CC (207, 71 bp). The 50-bp Ins/Del variant was amplified with specific primers and separated on a 3.5% agarose gel: Ins/Ins (297 bp), Ins/Del (297, 247 bp), Del/Del (247 bp).

**Results:**

Significant differences were found in the distribution of the *SOD1* 50-bp Ins/Del polymorphism, with the heterozygous I/D genotype more frequent in SCUD patients than controls (p < 0.05). No significant differences were observed for the +35A/C (exon 3/intron 3) polymorphism in terms of genotype or allele frequencies. Analysis of clinical parameters—including age at onset, disorder duration, polysubstance abuse, inpatient treatment, violent behavior, suicide attempts, psychotic symptoms, family history, and substance use frequency—showed no associations with genotype, except that patients with the AA genotype of +35A/C had a significantly higher prevalence of family psychiatric history (p < 0.05).

**Conclusions:**

Our study suggests that the *SOD1* 50-bp Ins/Del heterozygous (I/D) genotype may confer a potential disadvantage, as it was significantly more frequent in SCUD patients. The Del allele has been linked to decreased promoter activity of *SOD1*, which may reduce ROS detoxification and impair oxidative stress regulation. Given the strong interaction between ROS and DNA, this polymorphism could influence genome integrity and contribute to individual susceptibility to SCUD.

**Disclosure of Interest:**

None Declared

